# Multi-level evidence of titanium dioxide nanoparticle toxicity in blueberry plantlets

**DOI:** 10.1007/s12298-026-01773-9

**Published:** 2026-06-11

**Authors:** Viktor Husak, Ulyana Kilchytska, Ivan Myroniuk, Ihor Mykytyn, Volodymyr Lushchak, Alois Bilavcik, Olena Bobrova

**Affiliations:** 1 Department of Biochemistry and Biotechnology, Vasyl Stefanyk Carpathian National University, 57 Shevchenka Str., Ivano-Frankivsk, 76018 Ukraine; 2 Department of Chemistry, Vasyl Stefanyk Carpathian National University, 57 Shevchenka Str., Ivano-Frankivsk, 76018 Ukraine; 3 Research and Development University, Shota Rustaveli Str., Ivano-Frankivsk, 76018 Ukraine; 4 Czech Agrifood Research Center, Plant Physiology and Cryobiology Team, Drnovska 507/73, 16100 Prague, Czech Republic

**Keywords:** Blueberry plantlets, Titanium dioxide nanoparticles, Antioxidant enzymes, Oxidative stress, Nanotoxicology

## Abstract

Titanium dioxide nanoparticles (TiO_2_NPs) are increasingly proposed for agricultural applications; however, their crop-specific safety profiles remain insufficiently understood. Here, we provide, to our knowledge, the first integrative evidence that TiO_2_NPs exert predominantly negative effects on blueberry (*Vaccinium corymbosum* L.), a high-value woody perennial. In an in vitro 40-day exposure (30, 60, 90, 150 mg L^−1^), we quantified coordinated responses across physiological, histological, and biochemical levels. Shoot elongation declined at all doses (up to 57%), with reduced biomass at higher concentrations. Histological analysis showed progressive tissue disorganization, from mild cell compaction at low doses to necrosis and vascular damage at 150 mg L^−1^. Chlorophyll *a*, chlorophyll *b*, and carotenoid levels declined by up to 68%, indicating impaired photosynthetic capacity. Concurrently, TiO_2_NPs stimulated antioxidant defenses: phenolic and flavonoid content increased up to threefold, while superoxide dismutase (SOD) and ascorbate peroxidase (APX) activities rose by 138% and 147%, respectively. Correlation and sensitivity analyses confirmed strong antagonism between growth and defense responses. Shoot length positively correlated with pigment content (*r* = 0.99) but negatively with SOD (*r* = − 0.87) and flavonoids (*r* = − 0.75), suggesting a trade-off between growth and oxidative stress mitigation. SOD, APX, and carotenoids emerged as the most responsive biomarkers. Collectively, these multi-level data demonstrate that TiO_2_NPs inhibit development and disrupt tissue integrity in blueberry, supporting a precautionary perspective: TiO_2_NP-based fertilizers are not recommended for blueberry cultivation until safe exposure thresholds are validated under soil and field conditions.

**Supplementary Information:**

The online version contains supplementary material available at 10.1007/s12298-026-01773-9.

## Introduction

Titanium dioxide (TiO_2_), a widely studied metal oxide semiconductor, has gained significant attention due to its unique physicochemical properties, particularly in photocatalysis, photoprotection, and redox activity. TiO_2_ exists primarily in three polymorphic crystalline forms: anatase, rutile, and brookite, each of which differs in crystal structure, surface energy, and band gap characteristics (Eddy et al. 2023). Among them, anatase (with a band gap of ~ 3.2 eV) and rutile (~ 3.0 eV) are the most extensively explored, particularly for their photoactivity under ultraviolet (UV) light (Kovačič et al. 2022; Shand et al. 2013). Upon UV exposure, TiO_2_ nanoparticles (TiO_2_NPs) can generate of reactive oxygen species (ROS), including superoxide anions, hydroxyl radicals, and hydrogen peroxide, which can initiate redox reactions on plant and microbial surfaces (Jing et al. 2013; Ono and Iwahashi 2022). These properties have led to the widespread integration of TiO_2_NPs into diverse fields, ranging from environmental remediation and medicine to agriculture. Because TiO_2_NPs may function both as growth-promoting nano-agrochemicals and as photocatalytically active stressors capable of generating ROS, they represent an important model nanomaterial for evaluating the balance between beneficial and phytotoxic effects in crop systems.

In the agricultural sector, TiO_2_NPs have emerged as promising agents for enhancing plant growth, nutrient uptake, and stress tolerance (Farahi and Mostafa 2023). TiO_2_NPs are increasingly investigated for their potential to enhance plant tolerance to abiotic stresses, including temperature extremes, by modulating photosynthetic efficiency and antioxidant responses, although their effects remain dependent on particle properties and environmental conditions (Alabdallah et al. 2024). Previous studies have shown that nanoparticle size, surface area, crystallinity, and aggregation state strongly influence plant uptake, ROS generation, and phytotoxicity. At low concentrations, TiO_2_NPs can enhance light absorption efficiency, stimulate photosynthetic activity by promoting chlorophyll biosynthesis, positively influence pigment production, boost both enzymatic and non-enzymatic antioxidant activities, and facilitate nutrient mobilization at the root – soil interface (Babaei et al. 2025; Ko et al. 2019; Li et al. 2022; Lyu et al. 2017). Their redox activity may also support nitrate assimilation and delay senescence under suboptimal conditions. However, these beneficial effects are highly dose-dependent. When applied at excessive concentrations or under continuous exposure, TiO_2_NPs have been shown to induce oxidative stress in plants, causing membrane damage, pigment and protein degradation, and impairment of metabolic and signaling pathways (Chen et al. 2019; Szymanska et al. 2016). In such cases, plants respond by activating enzymatic and non-enzymatic antioxidant defenses, including superoxide dismutase (SOD), ascorbate peroxidase (APX), and polyphenolic compounds. The balance between stimulatory and inhibitory effects is often species-specific, necessitating careful evaluation under controlled conditions.

Despite growing research into plant–nanoparticle interactions, most published studies have focused on herbaceous species or agricultural model plants, such as *Oryza sativa* and *Triticum aestivum* (Chaudhary et al. 2020; Jiang et al. 2017; Phothi et al. 2020). Comparatively little is known about how TiO_2_NPs affect the physiology of woody perennials, especially high-value horticultural crops like blueberry (*Vaccinium corymbosum* L.). Blueberry is a perennial shrub cultivated globally for its antioxidant-rich fruits and nutraceutical properties (Ghosh et al. 2024). However, it is highly sensitive to environmental and chemical stressors, and its tissue culture systems provide an excellent model for studying abiotic stress effects on cellular organization, pigment stability, and biochemical adaptation (Correia et al. 2024). Understanding how TiO_2_NPs interact with blueberry tissues is essential, both from a crop management perspective and for assessing potential risks associated with nanoparticle exposure in agroecosystems.

In vitro culture platforms offer a tightly controlled environment for evaluating nanoparticle effects, minimizing external variability caused by soil composition, microbial communities, and fluctuating weather conditions (Jan et al. 2025; Kumari et al. 2024). This system allows researchers to precisely monitor early developmental changes, including shoot morphogenesis, leaf histoarchitecture, photosynthetic efficiency, and oxidative stress responses. Moreover, the combination of anatomical and biochemical assessments provides a more comprehensive view of how nanoparticles modulate both structural integrity and physiological resilience in plants.

In this study, we investigated the influence of different concentrations of TiO_2_NPs on growth regulation and stress physiology in *V. corymbosum* cultured in vitro. Specifically, we evaluated the effects of TiO_2_NPs on morphometric traits, leaf histoarchitecture, photosynthetic pigment levels, total phenolic and flavonoid content, and antioxidant enzyme activity. By integrating anatomical and biochemical markers, this work provides a multi-level assessment of TiO_2_NP phytotoxicity in blueberry under controlled in vitro conditions. To our knowledge, few studies have investigated TiO_2_NP-induced phytotoxicity in woody perennial fruit crops such as blueberry, particularly using an integrated physiological, histological, and biochemical approach. These findings extend current understanding beyond herbaceous model species and provide additional insight into species-specific responses and potential risks associated with nanoparticle use in precision agriculture.

## Materials and methods

### Characterization of titanium dioxide nanoparticles

TiO_2_NPs used in this study were synthesized and characterized by our research group, following the method outlined by Mironyuk et al. (2019, 2022). The synthesis was based on liquid-phase hydrolysis of an aqueous titanium complex derived from titanium tetrachloride (TiCl_4_). Specifically, the titanium precursor complex, [Ті(ОН_2_)_6_]^3+^·3Cl^−^, was prepared according to a previously described protocol.

For nanoparticle formation, the titanium aqua complex solution was diluted with distilled water to adjust the pH to between 0.5 and 2, then maintained at 60–70 °C under continuous stirring for 30–40 min. To initiate controlled hydrolysis and precipitation, sodium hydroxide solution was added dropwise at the same temperature until the pH reached 6–7. The resulting TiO_2_precipitate was then filtered under vacuum, thoroughly washed with distilled water to remove chloride ions, and dried at 120–140 °C, yielding a white, amorphous-to-nanocrystalline powder. The dried material was subsequently thermally treated to induce phase transformation into anatase TiO_2_, a photocatalytically active crystalline form preferred for plant–nanoparticle interaction studies due to its high surface reactivity and stability.

**Fig. 1:**
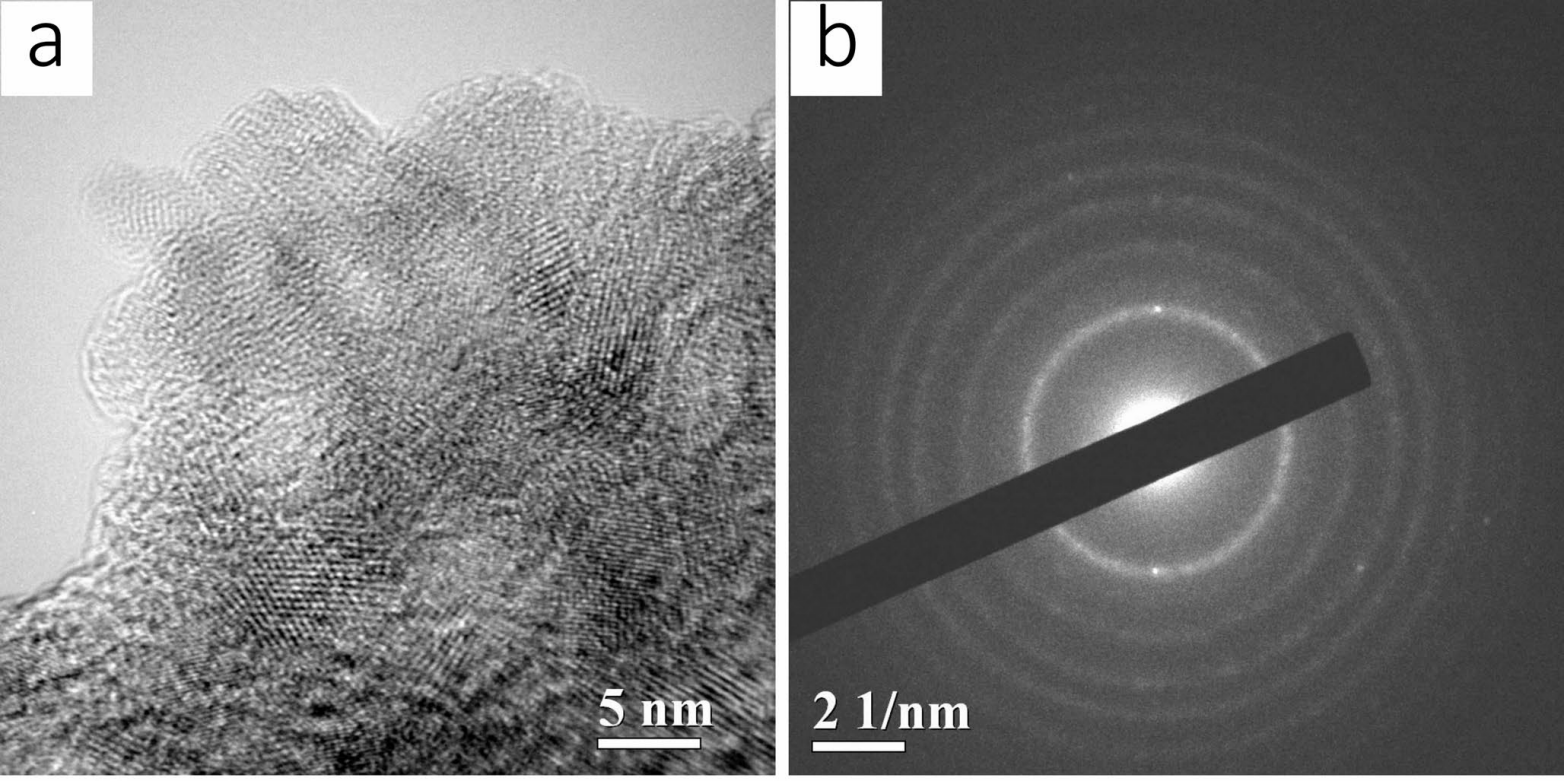
Transmission electron microscopy (TEM) characterization of titanium dioxide nanoparticles. **a** TEM image showing anatase-phase TiO_2_ nanocrystals with well-resolved lattice fringes; the average particle diameter was approximately 4–6 nm. **b** The selected area electron diffraction pattern displays concentric diffraction rings, confirming the nanocrystalline structure

To characterize the structural and morphological features of the synthesized TiO_2_ nanoparticles, transmission electron microscopy (TEM) was performed. TEM images (Fig. 1a) revealed uniform, nearly spherical nanoparticles with clearly resolved atomic lattice fringes, indicating high crystallinity. The average particle diameter was approximately 4–6 nm, based on measurements from over 100 individual particles. The selected area electron diffraction pattern (Fig. 1b) displayed concentric rings, which are indicative of the small particle diameter and confirm their nanocrystalline structure.

These nanoparticles were suspended in sterile distilled water and subjected to ultrasonic dispersion for 30 min before use, to ensure uniform distribution in the culture medium. The dispersed nanoparticles were then added to a culture medium at different concentrations as described in Sect.  2.2.

### In vitro culture of plant material and experimental design

The plant material used in this study consisted of sterile shoot explants of *V. corymbosum* cultivar ‘Spartan’, each approximately 1 cm in length. The explants were cultured under controlled conditions: a temperature range of 25–28 °C, under a photoperiod of 16 h of light and 8 h of darkness (50 µmol m^− 2^ s^− 1^). These parameters were maintained throughout the experimental period using a growth chamber optimized for micropropagation studies.

Woody Plant Medium (WPM) was utilized as the basal culture medium, formulated specifically for micropropagation of woody species (Lloyd and McCown 1981). The medium was supplemented with TiO_2_ nanoparticles at four concentrations: 30, 60, 90, and 150 mg L^− 1^. TiO_2_NPs were added to the medium using magnetic stirring, followed by autoclaving. A control group consisted of plants grown on Woody Plant Medium without adding TiO_2_NPs. After autoclaving, the medium was visually inspected before use. No gross precipitation was observed; however, because DLS/zeta analyses were not conducted after sterilization, nanoscale aggregation in the medium cannot be excluded.

Plant explants were cultured for 40 days. During this period, shoot regeneration was observed, particularly from axillary buds. These regenerated shoots were subsequently used for all analyses. Each treatment group included three biological replicates, with three individual plantlets per replicate (*n* = 9 per treatment). The overall experimental design enabled the evaluation of morphological, histological, and biochemical changes induced by nanoparticle exposure.

### Morphological analysis

At the end of the cultivation period (day 40), whole shoots were harvested from in vitro plants. For each treatment, three independent biological replicates were sampled; from each vessel, three randomly selected shoots were measured (total *n* = 9 per treatment). Immediately after removal from the culture medium, shoots were positioned flat without stretching. Length was measured along the main axis from the stem base at the medium surface to the shoot apex using a digital caliper (0.01 mm resolution). Two independent readings per shoot were recorded; if they differed by > 2%, a third reading was taken and the median retained. Since roots were absent, the entire shoot (stem + leaves) was used for mass determination. Samples were gently rinsed in distilled water to remove medium residues and blotted on lint-free paper for 2 min to remove surface moisture. Fresh weight was recorded on an analytical balance (0.01 mg resolution). Shoots exhibiting mechanical damage or severe hyperhydricity were excluded a priori. Instruments were zeroed before each session and checked with calibration standards. For each treatment, measurements were summarized at the biological-replicate level and then used for statistical analysis.

### Histological analysis of leaf tissues

For histological examination, leaf tissues from control and treated plants were collected and fixed in 10% neutral-buffered formalin for 24 h at room temperature. After fixation, samples were processed following standard histological protocols involving dehydration through a graded ethanol series, clearing with xylene, and embedding in paraffin wax.

Sections of 5 μm thickness were obtained using a rotary microtome and subsequently stained with hematoxylin and eosin to visualize tissue organization and anatomical structures. The staining protocol followed the method described by Luna (1968), with modifications adapted from Criswell et al. (2024). Microscopic analysis was performed under a compound light microscope to assess cellular integrity, mesophyll structure, and vascular tissue development.

### Preparation of extracts and supernatants

**Pigment extraction:** For pigment analysis, fresh leaf tissue was ground in chilled 96% ethanol at a tissue-to-solvent ratio of 1:10 (w/v), adding a small amount of calcium carbonate to stabilize pigments and prevent oxidation. The homogenate was centrifuged at 7000×g for 10 min at 4 °C to obtain the supernatant containing the pigments. The extraction procedure was repeated three times to ensure complete pigment recovery.

**Aqueous extract for phenolic and flavonoid analysis:** An aqueous extract was prepared to quantify total phenolic compounds and flavonoids. Precisely 50 mg of leaf tissue was weighed, placed into glass test tubes, and mixed with 0.5 mL of boiling distilled water. The mixture was then incubated in a water bath at 100 °C for 25 min in tightly sealed tubes to prevent evaporation. After cooling, the extract was filtered and stored at 4 °C until analysis.

**Homogenate preparation for enzyme assays:** Enzymatic analyses require fresh leaf homogenates. Approximately 100 mg of plant tissue was homogenized on ice in an extraction buffer at a 1:10 (w/v) ratio. The buffer consisted of 50 mM potassium phosphate buffer (pH 7.0), 0.5 mM EDTA, 0.8 mM phenylmethylsulfonyl fluoride, and distilled water. The mixture was centrifuged, and the supernatant was used immediately for enzyme activity measurements.

### Determination of leaf pigments

The optical density of the pigment-containing ethanol extract was measured using a ULAB 102 UV spectrophotometer. Absorbance readings were recorded at 664.2 nm (chlorophyll *a*), 648.2 nm (chlorophyll *b*), and 470 nm (carotenoids). To measure anthocyanin content, one drop of concentrated hydrochloric acid (37%) was added to each sample, and absorbance was recorded at 530 nm.

All readings were taken against a 96% ethanol blank. Concentrations of chlorophylls *a* and *b*, carotenoids, and anthocyanins were calculated using the equations described by Gitelson et al. (2006), which account for the overlapping absorbance spectra of these pigments.

#### Determination of total phenolics and flavonoids

**Flavonoid quantification:** Flavonoid content was determined using a colorimetric aluminum chloride method. Calibration standards were prepared using a 0.1% quercetin solution in varying volumes. Each reaction mixture included sodium nitrite (5%), aluminum chloride (10%), and 50 µL of either extract or standard. After incubation for 6 min, 1 M sodium hydroxide was added, and the total volume was adjusted to 2 mL with distilled water. Absorbance was measured at 510 nm (Ayele et al. 2022). Results were calculated from the quercetin standard curve and expressed as mg Eq QE gwm^− 1^ (milligrams of quercetin equivalents per gram of wet mass).

**Total phenolic content:** Total phenolic content was estimated using the Folin–Ciocalteu method. Calibration standards were prepared using gallic acid, while experimental samples contained 50 µL of extract. Each reaction mixture included diluted Folin–Ciocalteu reagent (1:10) and 7.5% sodium carbonate solution, with the total volume adjusted to 2 mL using distilled water. Samples were incubated at 45 °C for 15 min. Absorbance was measured at 765 nm. Concentrations were determined from the gallic acid standard curve (Sánchez-Rangel et al. 2013) and expressed as mg Eq GAE gwm^− 1^ (milligrams of gallic acid equivalents per gram of wet mass).

### Enzyme activity assays

**Superoxide dismutase (sOD**,** EC 1.15.1.1):** activity was measured using a photochemical method based on inhibition of Nitroblue Tetrazolium (NBT) reduction. The reaction mixture contained 2 µM riboflavin, 13 mM methionine, 63 µM NBT, 0.1 mM EDTA, and 50 mM potassium phosphate buffer (pH 7.8). Samples were placed in Eppendorf tubes and exposed to fluorescent light for 15 min to initiate the reaction, while control tubes (no enzyme) were kept in the dark. The degree of NBT reduction was assessed by measuring absorbance at 560 nm. One unit (U) of SOD activity caused 50% inhibition of the color change of the mixture reaction (Babitha et al. 2002).

**Ascorbate peroxidase (APX**,** EC 1.11.1.11):** activity was assessed by measuring the absorbance decrease due to ascorbate oxidation at 290 nm. The reaction mixture consisted of 50 mM potassium phosphate buffer (pH 7.0), 0.5 mM EDTA, 20 mM ascorbic acid, and 10 mM hydrogen peroxide. The molar extinction coefficient for ascorbic acid (2800 M^−1^ cm^−1^) was used to calculate enzyme activity in µmol ascorbate oxidized per minute per mg protein (Hiner et al. 2000). One U of APX activity was defined as the amount of enzyme consuming 1 µmol of substrate or generating 1 µmol of product per minute (U mg protein^− 1^).

The protein concentration was determined with Coomassie brilliant blue G-250 according to the method of Bradford (Bradford 1976) with bovine serum albumin as a standard.

### Statistical analysis

All experiments were conducted using three independent biological replicates, each consisting of three technical replicates (*n* = 9 per treatment). Results are presented as mean (M) ± standard error of the mean (SEM). All experimental data were subjected to one-way analysis of variance (ANOVA) followed by Dunnett’s post hoc test. Differences were considered statistically significant at *p* < 0.05. To ensure data integrity, outlier detection was conducted using both Chauvenet’s criterion and the Irwin test. Observations identified as statistical outliers by either method were excluded only if justified by experimental or technical inconsistencies. Statistical analyses were performed using R software (version 4.3.2; R Foundation for Statistical Computing, Vienna, Austria) and OriginPro 2021 software (OriginLab Corporation, Northampton, MA, USA), which were used for ANOVA computations, group comparisons, and graphical plotting of descriptive statistics.

In addition, Python 3.12 programming language was employed for advanced data visualization, including:

- The construction of correlation heatmaps to evaluate interrelationships between measured traits;
- Radar (spider) plots to represent percentage deviation from control across traits;
- And horizontal bar charts to visualize relative changes in key biomarkers under TiO_2_NPs exposure.

All custom scripts were developed using libraries including Pandas, NumPy, Matplotlib, and Seaborn.

## Results

### Effects of TiO_2_NPs on morphological traits of blueberry plantlets

**Fig. 2:**
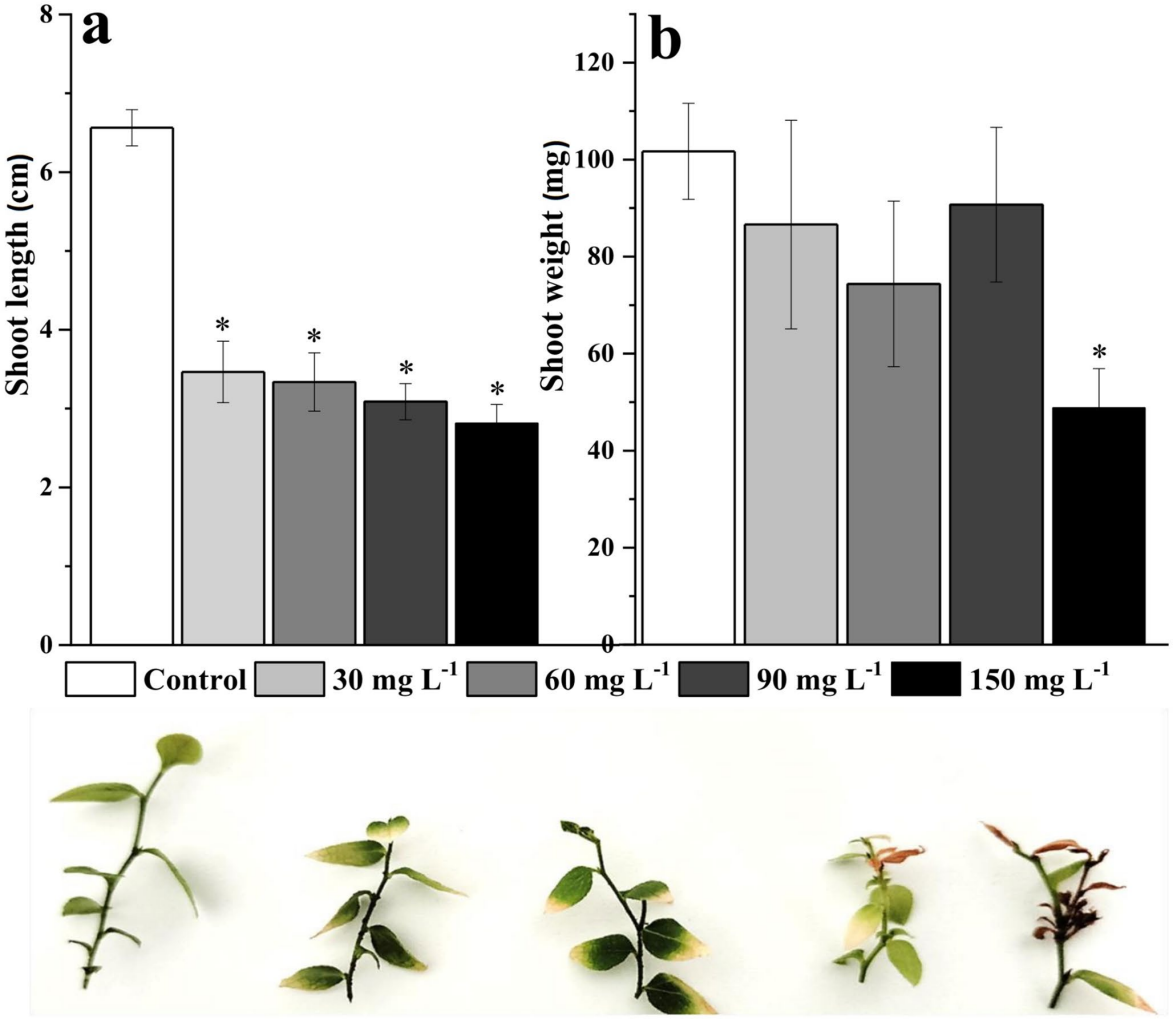
The effects of TiO_**2**_NPs (30, 60, 90, 150 mg L^− 1^) on shoot length (**a**) and weight (**b**) of in vitro blueberry plantlets cultivated in Woody Plant Medium during 40 days. Data are presented as means ± SEM, *n* = 6–9. *Significantly different from the control (without TiO_2_NPs) group of plants (*p* < 0.05) according to ANOVA followed by Dunnett’s test

The exposure of *V. corymbosum* plantlets to various concentrations of TiO_2_NPs (30, 60, 90, and 150 mg L^−1^) for 40 days in vitro resulted in a clear concentration-dependent reduction in shoot development and biomass accumulation. As illustrated in Fig. 2, shoot length significantly decreased at all tested concentrations of TiO_2_NPs. Specifically, the reduction in shoot length reached 47% at 30 mg L^−1^ and up to 57% at 150 mg L^−1^ compared to the control group (*p* < 0.05). Similarly, shoot weight showed a marked decrease, especially at 150 mg L^−1^, where it was approximately 50% lower than in untreated plants.

### Leaf anatomical modifications induced by TiO_2_NPs

**Fig. 3:**
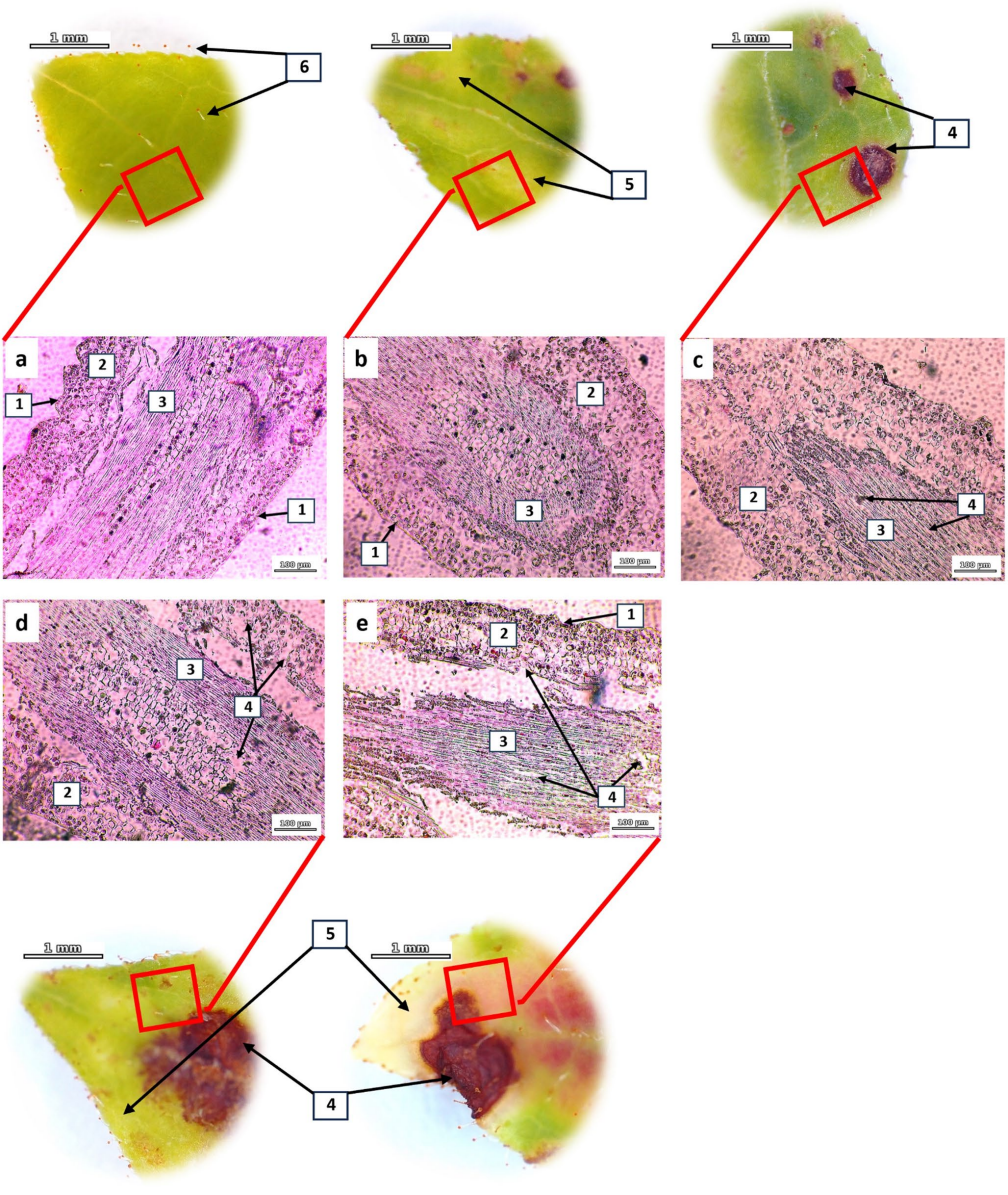
Morphological (15×) and histological (120×) characterization of blueberry leaves under the influence of different concentrations of TiO_2_NPs (**a** – control; **b** – 30 mg L^− 1^; **c** – 60 mg L^− 1^; **d** – 90 mg L^− 1^; **e** – 150 mg L^− 1^). 1 – epidermis; 2 – spongy parenchyma; 3 – palisade parenchyma; 4 – necrotic damage of tissues; 5 – chlorosis; 6 – trichomes

Histological examination of leaf tissues revealed progressive structural degradation with increasing TiO_2_NPs concentration (Fig. 3). In control plantlets (Fig. 3a), leaf tissue anatomy exhibited normal cellular organization with intact epidermal layers, well-developed palisade parenchyma consisting of elongated, tightly packed cells, and loosely arranged spongy mesophyll cells.

At 30 mg L^−1^ TiO_2_NPs (Fig. 3b), the leaf tissue remained relatively well-preserved, although slight compaction of palisade mesophyll cells and a marginal reduction in intercellular spaces within the spongy parenchyma were observed. These subtle changes may indicate an early stress response without substantial anatomical disruption.

At 60 mg L^−1^ TiO_2_NPs (Fig. 3c), more pronounced signs of cellular stress were evident. Partial collapse of palisade parenchyma cells, cytoplasmic shrinkage, and the formation of dark intracellular inclusions – possibly associated with nanoparticle accumulation or oxidative stress responses – were noted.

At 90 mg L^−1^ TiO_2_NPs (Fig. 3d), the severity of cellular damage increased. Structural deformation of vascular bundles, cytoplasmic aggregation, and degradation of mesophyll tissue integrity were frequently observed. Signs of cellular dehydration were apparent, and the architecture of the palisade layer appeared substantially compromised.

Tissue necrosis, cellular disintegration, and vascular deformation became increasingly pronounced with rising TiO_2_NP concentrations, particularly at 90 and 150 mg L^−1^, indicating severe structural disruption under high nanoparticle exposure. The most significant tissue destruction occurred at 150 mg L^−1^ (Fig. 3e), where massive cell lysis, extensive vacuolization, and necrotic zones were evident. The epidermis appeared collapsed in several regions, and vascular elements were visibly deformed.

### Effects of TiO_2_NPs on photosynthetic pigments

**Fig. 4:**
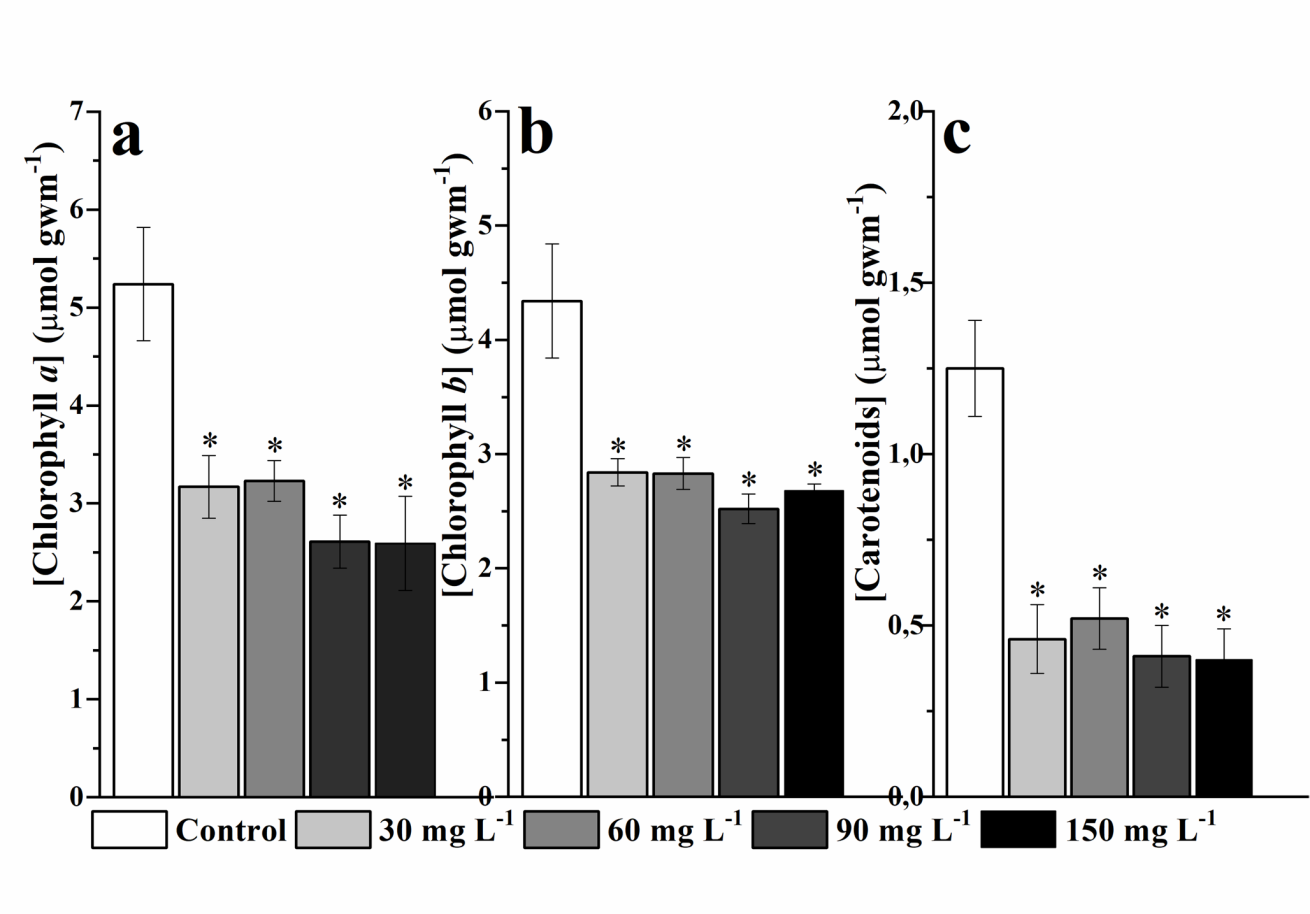
Concentrations of chlorophyll a (**a**), chlorophyll b (**b**), and carotenoids (**c**) in blueberry plantlets, exposed to different concentrations of TiO_2_NPs. Data are presented as means ± SEM, *n* = 6–8. *Significantly different from the control (without TiO_2_NPs) group of plants (*p* < 0.05) according to ANOVA followed by Dunnett’s test

Chlorophyll *a*, chlorophyll *b*, and carotenoid concentrations were significantly altered by TiO_2_NPs exposure (Fig. 4).

Chlorophyll *a* content decreased by 39%, 38%, 50%, and 51% at 30, 60, 90, and 150 mg L^−1^ of TiO_2_NPs, respectively, relative to the control (5.24 ± 0.58 µmol gwm^− 1^) (Fig. 4a). Chlorophyll *b* levels showed a parallel trend, declining by 34%, 35%, 42%, and 38% (Fig. 4b). The observed reductions were statistically significant (*p* < 0.05) and reflect the sensitivity of pigment biosynthesis or stability to TiO_2_NP-mediated oxidative stress.

Carotenoid levels were also markedly suppressed, with decreases of 63%, 58%, 67%, and 68% across the same treatment concentrations (Fig. 4c). These pigments are important not only for light harvesting but also for photoprotection and ROS scavenging, suggesting that nanoparticle exposure may interfere with redox homeostasis in chloroplasts.

Interestingly, the content of anthocyanins remained statistically unchanged across all treatments (data not shown).

### Enhancement of TiO_2_NPs phenolic compounds and flavonoids

**Fig. 5:**
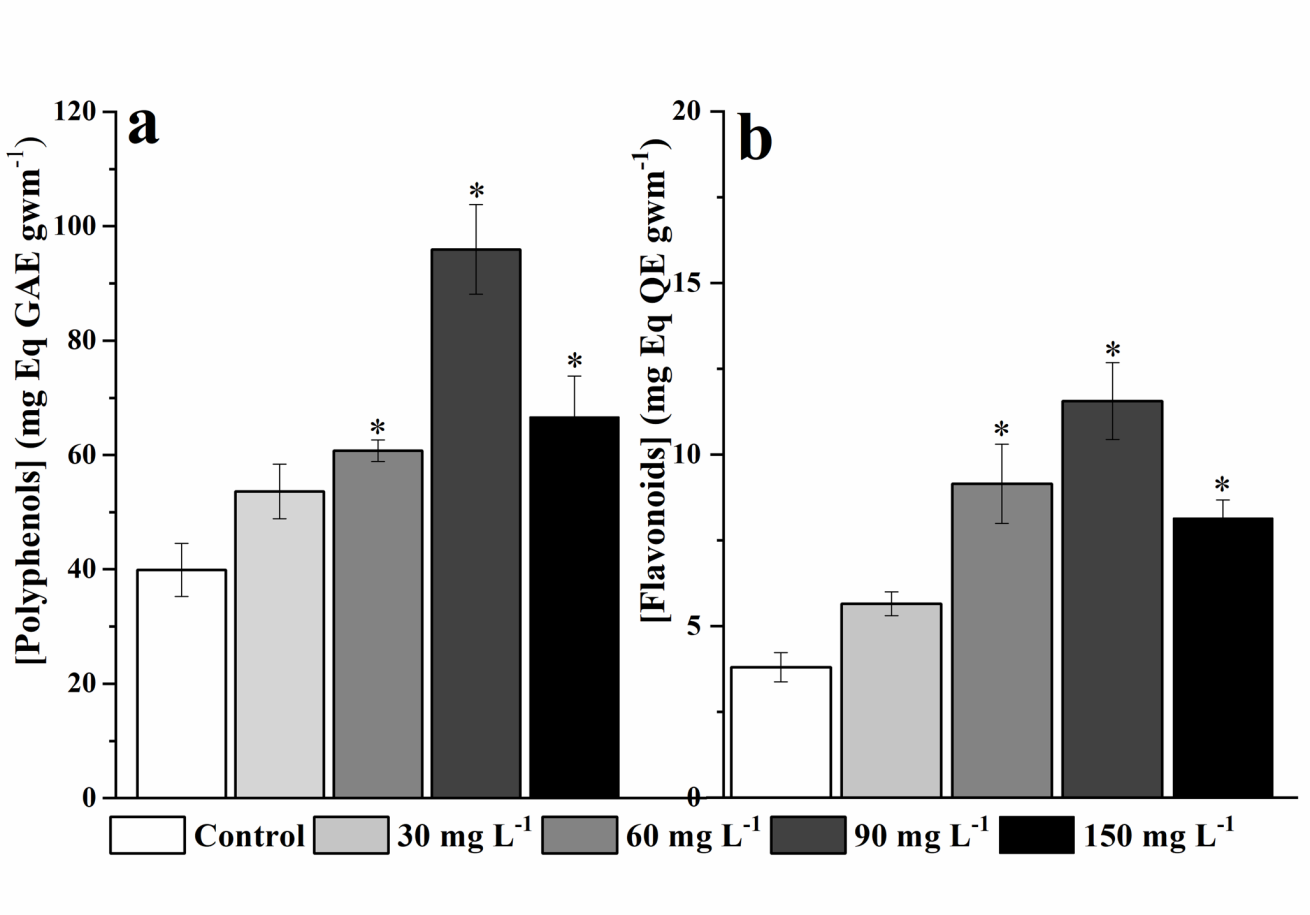
Amounts of total polyphenols (**a**) and flavonoids (**b**) of different plantlets of* V. corymbosum*, exposed to control conditions or 30, 60, 90, and 150 mg L^− 1^ of TiO_2_NPs for 40 days. Data are presented as mean ± SEM, *n* = 6–8. *Significantly different from the control (without TiO_2_NPs) group of plants (*p* < 0.05) according to ANOVA followed by Dunnett’s test

Biochemical assays revealed a significant increase in secondary metabolites in blueberry leaves treated with TiO_2_NPs (Fig. 5).

Аt TiO_2_NP concentrations of 60, 90, and 150 mg L^− 1^ in the cultivation medium, total phenolic content was by 52%, 141%, and 67% higher, respectively, as compared with the control plants (Fig. 5a). Flavonoid concentrations showed a similar pattern, increasing by approximately 141%, 204%, and 114% at the same concentrations, respectively, relative to the control (3.80 ± 0.43 mg Eq QE gwm^− 1^) (Fig. 5b).

### Effects of TiO_2_NPs on antioxidant enzymes

**Fig. 6:**
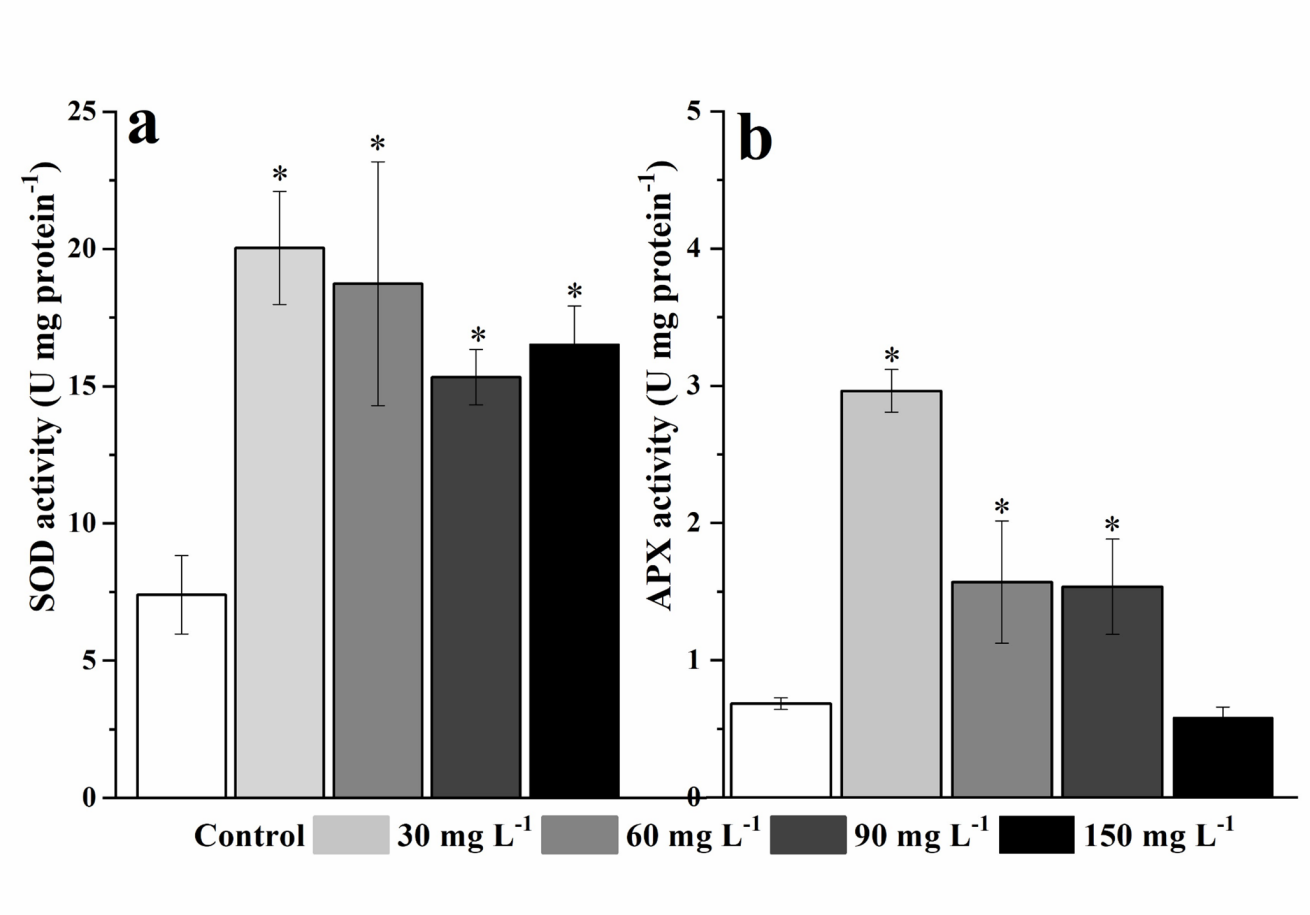
The activities of superoxide dismutase (SOD, U mg protein^− 1^) (**a**) and ascorbate peroxidase (APX, U mg protein^− 1^) (**b**) in blueberry plantlets, exposed to control conditions or 30, 60, 90, and 150 mg L^− 1^ of TiO_**2**_NPs for 40 days. Data are presented as mean ± SEM, *n* = 6–8. *Significantly different from the control (without TiO_2_NPs) group of plants (*p* < 0.05) using ANOVA followed by Dunnett’s test

Antioxidant enzyme activity was significantly modulated by TiO_2_ nanoparticle treatments, as shown in Fig. 6.

SOD activity was significantly higher in plantlets exposed to TiO_2_NPs at all tested concentrations compared with the control (7.40 ± 1.43 U mg protein^− 1^). Particularly, SOD activity in explants of plants treated with 30, 60, 90, and 150 mg L^−1^ TiO_2_NPs was higher than the control by 171%, 153%, 107%, and 123%, respectively (Fig. 6a).

APX activity followed a similar trend. At 30, 60, and 90 mg L^−1^ TiO_2_NPs, APX activity significantly exceeded the control value (0.683 ± 0.042 U mg protein^− 1^) by 335%, 130%, and 125%, respectively. However, at the highest concentration (150 mg L^−1^), no significant difference from the control value was observed (Fig. 6b), suggesting that oxidative stress at the highest TiO_2_NP concentration may have exceeded the capacity of the APX-dependent antioxidant response.

### Correlation analysis and sensitivity profiling

To elucidate the coordinated physiological and biochemical responses of *V. corymbosum* plantlets to TiO_2_NP exposure, we conducted an integrated correlation analysis together with sensitivity profiling. This approach enabled the identification of key interdependent traits, potential adaptive mechanisms, and the most responsive stress biomarkers under in vitro conditions.

**Fig. 7:**
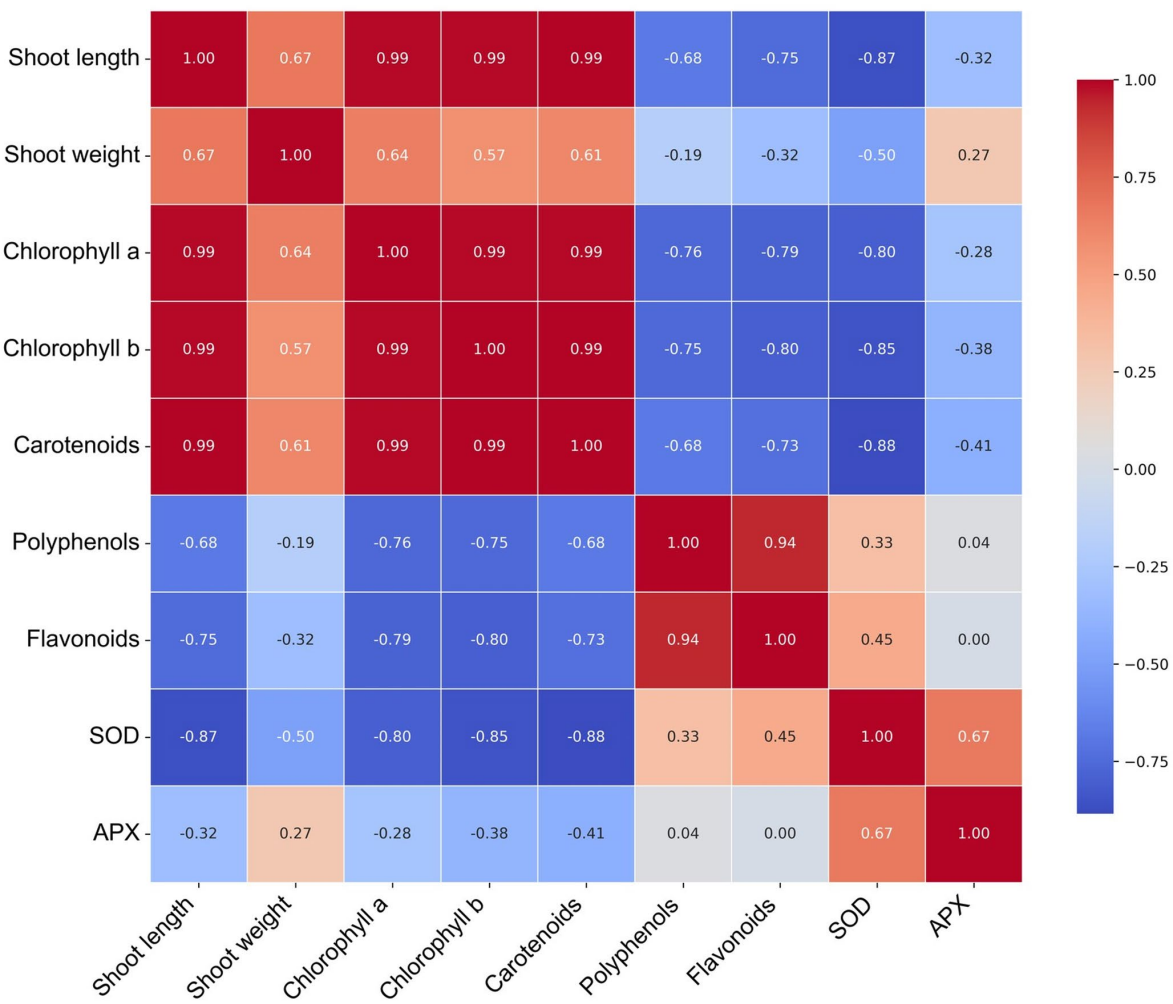
Correlation matrix of physiological and biochemical parameters of blueberry plantlets exposed to different concentrations of TiO_2_NPs. The matrix shows Pearson’s correlation coefficients between shoot growth traits (length and weight), photosynthetic pigments (chlorophyll a, chlorophyll b, carotenoids), and antioxidant responses (polyphenols, flavonoids, superoxide dismutase (SOD), and ascorbate peroxidase (APX)) following 40 days of in vitro exposure to TiO_2_NPs (0–150 mg L^− 1^). Darker red shades indicate strong positive correlations, whereas darker blue shades represent strong negative associations between the measured traits

A comprehensive Pearson correlation matrix was generated and visualized as a heatmap (Fig. 7, Table S1) to assess statistical relationships among measured parameters, including shoot length, biomass, photosynthetic pigment levels, antioxidant enzyme activities, and concentrations of phenolic compounds. The heatmap revealed a distinct pattern of associations that reflect how increasing concentrations of TiO_2_NPs influence multiple aspects of plant physiology.

Strong positive correlations were observed between shoot length and photosynthetic pigments – chlorophyll *a* (*r* = 0.99), chlorophyll *b* (*r* = 0.99), and carotenoids (*r* = 0.99) – suggesting a close dependency between growth performance and pigment integrity. This indicates that growth inhibition under TiO_2_NPs stress is closely tied to disruptions in chlorophyll biosynthesis and light-harvesting capacity.

In contrast, shoot length was negatively correlated with antioxidant-related traits such as SOD activity (*r* = − 0.87), total flavonoids (*r* = − 0.75), and polyphenols (*r* = − 0.68). These antagonistic relationships suggest a physiological trade-off in which the plant reallocates metabolic resources toward defense activation at the expense of vegetative growth. Such trade-offs are commonly observed in plants exposed to abiotic stress.

Moreover, positive correlations among antioxidant traits were also prominent: flavonoids and polyphenols displayed a strong association (*r* = 0.94), indicating coordinated regulation of non-enzymatic ROS scavenging systems. Similarly, SOD and APX activities were positively linked (*r* = 0.67), supporting coordinated enzymatic activity in ROS detoxification pathways. Interestingly, both chlorophyll a and b were negatively correlated with SOD (*r* = − 0.80 and − 0.85, respectively), further confirming the stress-induced trade-off between photosynthetic efficiency and oxidative stress management.

**Fig. 8:**
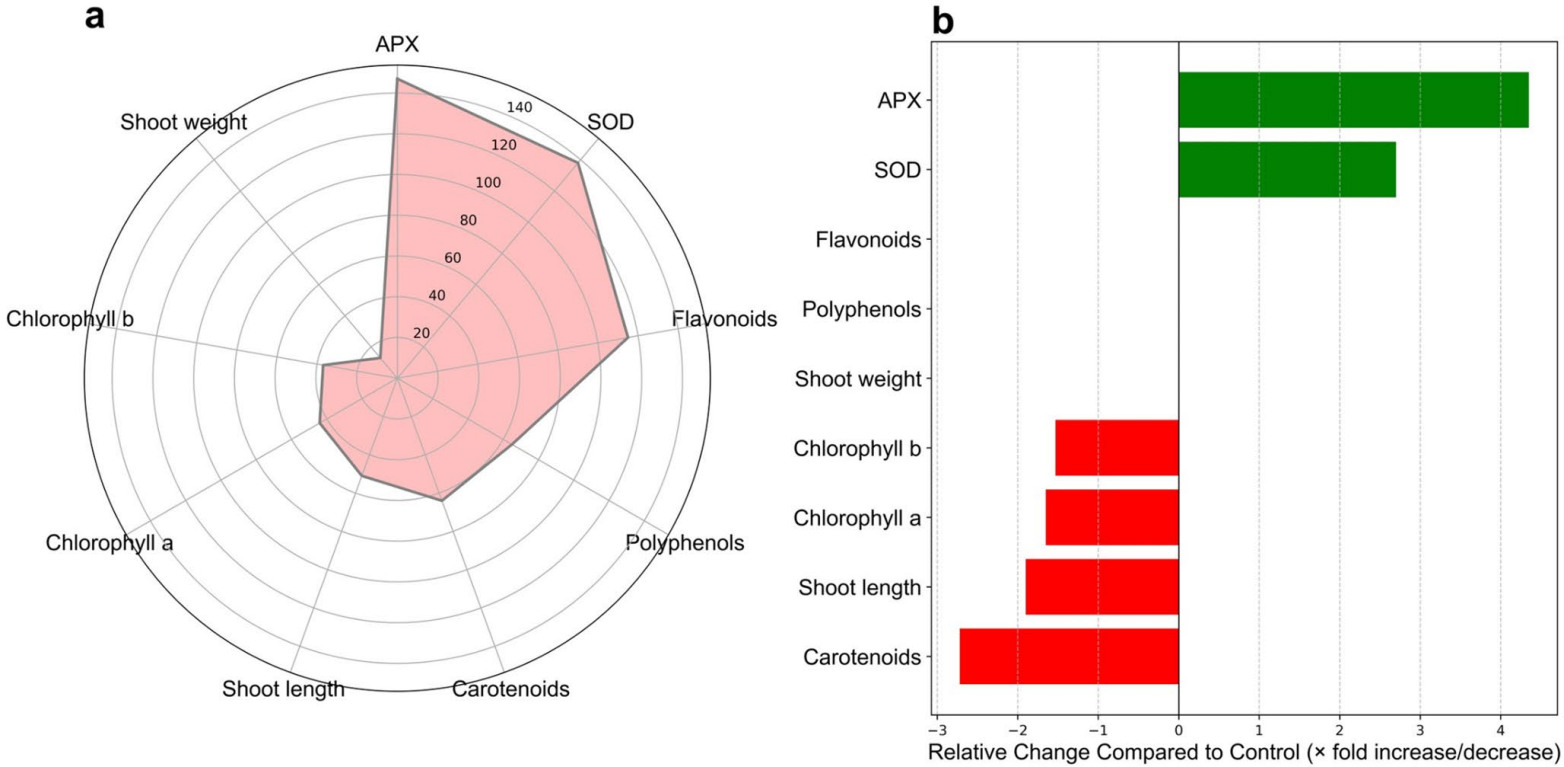
Sensitivity profiling of physiological and biochemical parameters of *V. corymbosum* under TiO_2_NPs exposure. **a** Radar chart showing the average percentage deviation of growth, pigment content, and antioxidant traits relative to the control across all TiO_2_NPs treatments. **b** Horizontal bar chart illustrating the relative change (fold increase or decrease) in individual plant parameters under 30 mg L^−1^ TiO_2_ nanoparticle treatment, highlighting the most responsive biomarkers (SOD, APX, and carotenoids)

To quantify the magnitude of trait responsiveness, a sensitivity profile was constructed based on the average percentage change from the control values (Fig. 8a). This radar plot identified APX (+ 147%), SOD (+ 138%), and flavonoids (+ 115%) as the most reactive indicators of TiO_2_NPs-induced oxidative stress. Moderate changes were observed in polyphenol content (+ 65%), while chlorophyll *a* (–44%), chlorophyll *b* (–37%), carotenoids (–64%), and shoot length (–51%) showed marked declines, reflecting structural and functional damage. Shoot biomass exhibited the least sensitivity (–13%), possibly due to delayed mass accumulation under early stress stages.

To capture early physiological responses, a bar plot representing parameter changes at the lowest TiO_2_NP concentration (30 mg L^−1^) was also generated (Fig. 8b). While shoot length displayed a 1.9-fold decrease, shoot weight remained relatively stable. Pigment loss was evident with decreases in chlorophyll *a* (1.6-fold), chlorophyll *b* (1.5-fold), and carotenoids (2.7-fold). Enzyme activities, however, were sharply elevated – SOD (2.7-fold) and APX (4.3-fold) – indicating rapid activation of ROS-detoxifying pathways. Phenolic and flavonoid levels remained statistically unchanged at this concentration, suggesting that prolonged or higher-dose exposure may be necessary for full induction of secondary antioxidant mechanisms.

Altogether, the correlation and sensitivity profiling demonstrated a multifaceted stress response where TiO_2_NPs exposure leads to pigment degradation, suppressed shoot elongation, and pronounced activation of enzymatic antioxidant defenses. These results identify carotenoids, SOD, and APX as sensitive biomarkers for detecting and quantifying nanoparticle-induced physiological stress in *V. corymbosum* cultured in vitro.

## Discussion

This work addresses a critical gap in nanoparticle risk assessment for woody perennial fruit crops by delivering, to our knowledge, the first comprehensive demonstration of TiO_2_NP-induced phytotoxicity in blueberry across physiological, histological, and biochemical domains.

A key observation was the consistent suppression of shoot elongation across all tested TiO_2_NP concentrations. Shoot length reductions of up to 57% suggest early sensitivity to nanoparticle exposure, aligning with findings in *Mentha piperita* (Samadi et al. 2014), *Spirodela polyrrhiza* (Movafeghi et al. 2018), and *Triticum aestivum* (Jiang et al. 2017), where reduced elongation is linked to impaired photosynthesis, disrupted water transport, hormonal imbalance, and oxidative stress. The relatively smaller change in shoot weight compared to length highlights a potential lag in biomass response or compensation mechanisms buffering mass accumulation.

Histological analysis revealed structural damage to leaf tissue in a concentration-dependent manner, ranging from mild compaction of parenchyma at low doses to severe cellular disintegration and vascular necrosis at higher concentrations. These findings mirror previous ultrastructural observations in *Solanum lycopersicum* (Tighe-Neira et al. 2024) and *Halophila stipulacea* (Mylona et al. 2020), where TiO_2_NPs caused chloroplast collapse and disrupted cellular integrity. The presence of cytoplasmic inclusions and vacuolation may indicate TiO_2_NP accumulation or stress-induced deposits (Mukherjee et al. 2020), potentially associated with ROS bursts or metabolic disturbance.

Physiologically, a pronounced decline in chlorophyll *a* and *b*, as well as carotenoids, was recorded, confirming TiO_2_-induced inhibition of pigment biosynthesis or enhanced degradation. These findings are consistent with previous studies demonstrating that TiO_2_NPs interfere with chloroplast structure and pigment biosynthesis, as reported in *Chlorella pyrenoidosa* (Middepogu et al. 2018), *Scenedesmus obliquus* (Li et al. 2020), *Trigonella foenum-graecum* (Missaoui et al. 2017), *Dunaliella salina*, and *Dunaliella tertiolecta* (Ghazaei and Shariati 2020). Similar pigment loss has also been observed in *Arabidopsis thaliana* (Ze et al. 2011) and *Chlamydomonas reinhardtii* (Chen et al. 2012). These reductions likely stem from damage to chloroplast membranes and impaired synthesis pathways under oxidative stress. Interestingly, anthocyanin content remained unchanged, potentially due to the short exposure period or the specific stress signature of TiO_2_NPs, which may not trigger anthocyanin biosynthesis in this species under in vitro conditions.

Elevated levels of phenolic compounds and flavonoids indicate activation of the non-enzymatic antioxidant defense, limiting ROS-mediated damage. Similar polyphenolic responses to TiO_2_NP exposure have been documented in *Salvia officinalis*, *Saponaria officinalis*, and *Cicer arietinum* (Ghorbanpour 2015; Hedayati et al. 2022; Ghorbani et al. 2023). Phenolics act as efficient ROS scavengers, while flavonoids further stabilize membranes and modulate redox-sensitive signaling. Together, these findings support a model in which TiO_2_NPs elicit oxidative and/or UV-related stress that activates phenylpropanoid metabolism, thereby enhancing antioxidant metabolite biosynthesis and helping restrain ROS accumulation in exposed tissues.

Enzymatic antioxidant activity mirrored these changes, with SOD and APX levels significantly elevated, especially at lower concentrations. This suggests an early activation of enzymatic ROS detoxification systems, aligning with findings in *Melissa officinalis* (Mohammadi et al. 2023). At the highest TiO_2_NP concentration, the decline in APX activity may suggest that oxidative stress exceeded the antioxidant capacity of the tissue, leading to partial enzyme inactivation, substrate limitation, and cellular damage that impaired APX-mediated detoxification, thereby reflecting a dose-dependent shift from adaptive to damaging effects.

Correlation analyses provided further insights, showing that shoot length was strongly positively correlated with pigment content and negatively correlated with antioxidant traits. This highlights a common trade-off in plant stress physiology: energy resources are diverted from growth and photosynthesis toward defensive responses, as also reported in *Calendula officinalis* (Lashkary et al. 2021) and *Cicer arietinum* (Ghorbani et al. 2023).

Conversely, shoot length was negatively correlated with SOD activity, flavonoids, and polyphenols, suggesting that under TiO_2_ stress, growth is compromised in favor of activating antioxidative defenses. Similar antagonistic relationships between growth inhibition and antioxidant induction have been described for many plant species under nanoparticle exposure (Lashkary et al. 2021; Hedayati et al. 2022; Ghorbani et al. 2023). Among antioxidant-related traits, flavonoids and polyphenols displayed a very strong positive correlation, implying coordinated non-enzymatic ROS-scavenging activity. SOD and APX activities were also positively correlated, reflecting enzymatic cooperation in detoxification pathways, in line with observations from Kumar et al. (2023). It suggests a synergistic network between enzymatic and non-enzymatic defenses, consistent with integrated ROS scavenging systems described by Mittler et al. (2011) and Farmer and Mueller (2013). Interestingly, both chlorophyll *a* and *b* were negatively correlated with SOD, suggesting antagonistic regulation between pigment stability and enzymatic stress responses. A similar trade-off has been reported in *Mentha arvensis* exposed to TiO_2_NPs (Kumar et al. 2023). Likewise, carotenoids showed positive correlation with chlorophylls and shoot length but a negative correlation with SOD, supporting their role as early markers of oxidative stress (Kumar et al. 2023).

Overall, the data suggest a stress-adaptation trade-off, where shoot elongation and photosynthesis are suppressed, while antioxidant systems are upregulated in response to oxidative stress induced by TiO_2_NPs (Missaoui et al. 2017; Hedayati et al. 2022; Ghorbani et al. 2023; Kumar et al. 2023).

Sensitivity analysis via radar plots confirmed the high reactivity of APX, SOD, and flavonoids. These traits respond rapidly to oxidative stimuli and are valuable indicators for nanotoxicity screening (Silva et al. 2019; Wang et al. 2021; Ghorbani et al. 2023). By contrast, growth traits and pigment levels exhibited more moderate shifts, which could reflect cumulative or downstream responses. Carotenoids, in particular, showed intermediate responsiveness, supporting their dual role as photoprotective pigments and antioxidants.

At the early exposure concentration of TiO_2_NPs, clear increases in enzymatic antioxidant activity were observed, while levels of flavonoids and phenolics remained relatively unchanged (Fig. 8b). Concurrently, chlorophyll *a* and *b* contents declined, along with a marked reduction in carotenoids, indicating that TiO_2_ interferes with pigment stability and overall photosynthetic efficiency, as reported in earlier studies (Szymańska et al. 2016; Kumar et al. 2023). These findings support the hypothesis that enzymatic antioxidant systems, such as superoxide dismutase and ascorbate peroxidase, act as the primary defense mechanism in the early phase of nanoparticle-induced stress. In contrast, non-enzymatic pathways involving secondary metabolites may be activated only under prolonged or more intense exposure (Mittler et al. 2011). This staggered defense response highlights the temporal hierarchy in plant stress adaptation and offers valuable insight into how physiological priorities are reorganized under abiotic challenge – a principle that could be leveraged in designing more resilient plant varieties.

Recent studies on berry crops have also demonstrated species-dependent physiological and biochemical responses to metal nanoparticles under environmentally relevant conditions. For example, Peshkova et al. (2024) reported nanoparticle accumulation patterns and altered antioxidant activity in *Vaccinium myrtillus* exposed to Ag and Cu nanoparticles under field conditions, supporting the importance of nanoparticle concentration, translocation behavior, and oxidative stress responses in perennial berry species.

In sum, our findings support a biphasic model of TiO_2_NP-induced stress in *V. corymbosum*: initial signaling via ROS triggers antioxidant defenses, followed by structural and metabolic consequences at higher exposures. This tiered response framework aligns with the “oxidative stress tolerance threshold” hypothesis proposed in recent nanotoxicology literature (Tan et al. 2018; Lushchak 2014). These insights are particularly valuable given the limited data on woody perennial species like blueberry, which differ substantially in their stress physiology compared to model crops.

The integrative approach employed here, which combines histology, physiology, biochemistry, and multivariate statistics, offers a comprehensive view of nanoparticle impacts on plant systems. This type of multidisciplinary investigation is increasingly recommended by researchers in the field of nanoecotoxicology (Farré et al. 2009; Tan et al. 2018). Future research should explore long-term exposure, field relevance, soil-nanoparticle interactions, and potential mitigation strategies (e.g., nanoparticle coatings, dosage control) to ensure safe and effective use of TiO_2_NPs in agriculture. Further molecular studies are warranted to dissect specific gene expression patterns associated with stress signaling, antioxidant activation, and tissue remodeling in response to engineered nanomaterials.

Several limitations of the present study should be acknowledged. First, nanoparticle characterization was based primarily on TEM observations, while dynamic light scattering (DLS) and zeta potential analyses were not performed. Therefore, hydrodynamic particle size distribution and colloidal stability in the culture medium could not be quantitatively assessed. Second, direct measurements of titanium uptake, accumulation, and tissue localization in blueberry explants were beyond the scope of this work. Consequently, the present study cannot distinguish between surface-associated and internalized TiO_2_NPs. Third, bulk TiO_2_ and ionic titanium controls were not included; therefore, nanoparticle-specific effects cannot be fully separated from general titanium-associated responses. In addition, because the experimental system relied on in vitro shoot explants lacking fully developed roots, root-mediated uptake and root-to-shoot translocation processes could not be evaluated. Finally, the results were obtained under controlled in vitro conditions, which may not fully reflect nanoparticle behavior and plant responses under greenhouse or field environments. Future studies integrating physicochemical nanoparticle characterization, elemental mapping, comparative titanium controls, and soil-based exposure systems will help further clarify the mechanisms and environmental relevance of TiO_2_NP-induced phytotoxicity in woody perennial crops.

## Conclusions

By integrating physiological, histological, and biochemical analyses in a woody perennial crop species, this study provides a comprehensive assessment of TiO_2_NP-induced stress responses in in vitro-cultured blueberry plantlets. The results demonstrate that TiO_2_NP exposure exerts dose-dependent and multifaceted effects, including growth inhibition, tissue disorganization, pigment degradation, and activation of antioxidant defense mechanisms. Exposure to higher TiO_2_NP concentrations significantly reduced shoot length and biomass, accompanied by progressive damage to mesophyll and vascular tissues. Simultaneously, marked declines in chlorophyll a, chlorophyll b, and carotenoid contents indicated impaired chloroplast function and photosynthetic efficiency. Despite these adverse effects, blueberry plantlets activated both enzymatic and non-enzymatic antioxidant defense systems. Increased levels of phenolic compounds and flavonoids, together with elevated superoxide dismutase and ascorbate peroxidase activities, reflected activation of oxidative stress response. Correlation analysis revealed a trade-off between growth and defense processes, as enhanced antioxidant activity was negatively associated with shoot growth and pigment stability. Sensitivity profiling further identified APX, SOD, and carotenoids among the most responsive biomarkers of early TiO_2_NP-induced stress under controlled in vitro conditions. Overall, the findings suggest that while lower TiO_2_NP concentrations may stimulate mild adaptive defense responses, higher concentrations induce physiological disruption and structural damage in blueberry tissues. These results highlight the importance of careful nanoparticle dosage management and emphasize the need for future studies incorporating long-term exposure, soil-based systems, nanoparticle uptake analyses, and comparative nano-versus-bulk titanium controls to better evaluate the environmental and agronomic relevance of TiO_2_NP applications in perennial crop systems.

## Supplementary Information

Below is the link to the electronic supplementary material.

Supplementary Material 1

## Data Availability

The data generated and/or analyzed during the current study are included in this published article. Additional datasets supporting the findings of this study are available from the corresponding author upon reasonable request.
