# Supplementary material for: Multi-level evidence of titanium dioxide nanoparticle toxicity in blueberry plantlets
Source: Physiol Mol Biol Plants. 2026 Jun 11;32(7):1655–70. doi: 10.1007/s12298-026-01773-9 (PMC13350783; doi:10.1007/s12298-026-01773-9)
Supplement: Supplementary file 1 — Supplementary Material 1 [file 12298_2026_1773_MOESM1_ESM.docx]

**Table S1.** Correlation matrix of physiological and biochemical parameters of blueberry plantlets exposed to different concentrations of titanium dioxide nanoparticles. The matrix shows Pearson’s correlation coefficients between shoot growth traits (length and weight), photosynthetic pigments (chlorophyll *a*, chlorophyll *b*, carotenoids), and antioxidant responses (polyphenols, flavonoids, superoxide dismutase (SOD), and ascorbate peroxidase (APX)) following 40 days of *in vitro* exposure to TiO₂NPs (0–150 mg L^-1^). Darker red shades indicate strong positive correlations, whereas darker blue shades represent strong negative associations between the measured traits.

|  | Shoot length | Shoot weight | Chlorophyll *a* | Chlorophyll *b* | Carotenoids | Polyphenols | Flavonoids | SOD | APX |
| --- | --- | --- | --- | --- | --- | --- | --- | --- | --- |
| Shoot length | 1.00 | 0.67 | 0.99 | 0.99 | 0.99 | -0.68 | -0.75 | -0.87 | -0.32 |
| Shoot weight | 0.67 | 1.00 | 0.64 | 0.57 | 0.61 | -0.19 | -0.32 | -0.50 | 0.27 |
| Chlorophyll a | 0.99 | 0.64 | 1.00 | 0.99 | 0.99 | -0.76 | -0.79 | -0.80 | -0.28 |
| Chlorophyll *b* | 0.99 | 0.57 | 0.99 | 1.00 | 0.99 | -0.75 | -0.80 | -0.85 | -0.38 |
| Carotenoids | 0.99 | 0.61 | 0.99 | 0.99 | 1.00 | -0.68 | -0.73 | -0.88 | -0.41 |
| Polyphenols | -0.68 | -0.19 | -0.76 | -0.75 | -0.68 | 1.00 | 0.94 | 0.33 | 0.04 |
| Flavonoids | -0.75 | -0.32 | -0.79 | -0.80 | -0.73 | 0.94 | 1.00 | 0.45 | 0.00 |
| SOD | -0.87 | -0.50 | -0.80 | -0.85 | -0.88 | 0.33 | 0.45 | 1.00 | 0.67 |
| APX | -0.32 | 0.27 | -0.28 | -0.38 | -0.41 | 0.04 | 0.00 | 0.67 | 1.00 |
